# Medical News and Miscellany

**Published:** 1898-07

**Authors:** 


### Medical News and Miscellany.

**For Sale,** very cheap, a Nedofik operating sofa, the same as used by Dr. Wyeth and in the Polyclinic. Address this office.

---

**Dr. T. S. Kennedy** has resigned the position of president of the Louisiana State Board of Medical Examiners.

---

**Dr. J. T. Eskridge,** of Denver, is taking a recreation and outing at Galveston and other cities on the gulf where fish are plenty.

---

**Announcement.**—Philadelphia, 1012 Walnut Street, June 30, 1898.—The partnership hitherto existing between Presley Blakiston and Kenneth M. Blakiston, under the firm name of P. Blakiston, Son & Co., expired June 30, 1898, on account of the death of the senior member.

The business of publishing, importation, and dealing in medical and scientific books, as established in 1843, will be continued by Kenneth M. Blakiston, trading as P. Blakiston's Son & Co.

**Dr. E. N. Shaw** of Cameron, has been selected as the surgeon of the 4th Texas Infantry (volunteers under the president's second call) and Drs. T. J. Halsell of Fort Worth, and ——— —— of Mexia, assistant surgeons.

---

**Dr. J. H. Moody,** of Mexia, Texas, a graduate of the Medical Department, Tulane University, New Orleans, has been appointed Assistant Physician of the Texas State Lunatic Asylum at Austin, vice Dr. Callahan, gone to the war.

---

**The Medical Board** to examine Texas recruits under the president's second call, are Dr. Bacon Saunders, Fort Worth, Dr. C. M. Rossen, Dallas, and Dr. Foscue, Waco. They receive $10 a day and traveling expenses when on duty.

---

**Dr. H. A. West,** of Galveston, late Professor of Medicine in the Texas Medical College and the popular secretary of the State Medical Association, was elected one of the Vice-Presidents of the American Medical Association at the Denver meeting.

---

**That's It.**—A subscriber who begun with Vol. 1 No. 1, in July, 1885, in renewing his subscription for the *fourteenth year* writes: "Times are not what they used to be, and I am dropping some of my journals, *but I cannot be without the 'Old Reliable,'—The breezy Red Back.*"

---

**New Journal.**--We have received Vol. I, No. 1, of the *Memphis Lancet.* Ten editors and one editorial in the initial number would indicate that they are to take it turn about. Good arrangement; it lets a fellow out nine months to rest his brain after the tax of getting up an editorial!

---

**Mellins' Food Company.**—The old reliable name of Doliber Goodale Co., so well and favorably known to every one who has used Mellins' Food (and who has not?), is changed to "The Mellins' Food Company." The address is the same as heretofore, 291 Atlantic Ave., Boston, Mass.

---

**Dr. Nicholos Senn** who has received the appointment of assistant surgeon general, U. S. A., has been sent to Cuba in charge of some twenty additional medical officers whose services, Gen. Shafter says, are badly needed. That is right. Dr.

Senn is just the man to be in charge of the wounded in Cuba; he is a military surgeon by instinct and training, and has written upon the subject I believe.

**The Louisiana State Board of Health** has established temporary quarantine against the coast of Mississippi, which includes Hancock, Jackson and Harrison counties, owing to the appearance of yellow fever at McHenry, Miss., one of the infected points of last year, a small saw-mill town. The quarantine, we understand, will be enforced until all danger of infection is passed.

The first cases, seven in number, were reported on June 9. There have been, in all, up to time of going to press, twenty-three cases. There are now only three cases in existence, of which two are convalescing, and the type has been mild, no deaths having occurred.

Persons from the quarantine counties can enter the State of Louisiana only after disinfection and ten days' detention at Camp Fontainebleau or in any city north of the fever region, the nearest probably being Atlanta.

These preventive measures seem harsh to those of our people who would like to go back and forth between New Orleans and the gulf coast resorts, but they must be deemed wise on the part of our State Board until further developments can be ascertained.—*New Orleans Medical and Surgical Journal.*

The New Orleans Board is Texas' outpost; as long as the above is in operation it is not necessary to quarantine our eastern border. The Governer issued his general proclamation on May 1st against all infected places. The citizens of an infected town are quarantined, they are not allowed to go anywhere; something that seems to be not generally understood.

**The First Nathan Lewis Hatfield Prize for Original Research in Medicine.**—The College of Physicians of Philadelphia announces through its committee that the sum of five hundred dollars will be awarded to the author of the best essay in competition for the above prize.

Subject: "A Pathological and Clinical Study of the Thymus Gland and its Relations."

Essay must be submitted on or before January 1st, 1900.

Each essay must be typewritten, designated by a motto or device, and accompanied by a sealed envelope bearing the same motto or device and containing the name and address of the author. No envelope will be opened except that which accompanies the successful essay.

The committee will return the unsuccessful essays if reclaimed by their respective writers or their agents within one year.

The committee reserve the right not to make an award if no essay submitted is considered worthy of the prize.

The treatment of the subject must, in accordance with the conditions of the trust, embody original observations or researches or original deductions.

The competition shall be open to members of the medical profession and men of science in the United States.

The original of the successful essay shall become the property of the College of Physicians.

The trustees shall have full control of the publication of the memorial essay. It shall be published in the Transactions of the College, and also when expedient as a separate issue.

Address, J. C. Wilson, M. D., Chairman,
College of Physicians, 219 South Thirteenth Street, Philadelphia, Pa.

---

**Morphine Anesthesia.**—Dr. John A. Wyeth, in speaking of anesthetics, says: "The use of morphine as an anesthetic agent has not received the attention it deserves. I removed a larnyx on one occasion without the use of a trachea tube and with complete anesthesia, by producing a profound narcosis by the hypodermic use of morphine. I gave the patient two ounces of whiskey half an hour before, and repeated this five minutes before operation; twenty minutes before operation one-quarter of a grain of morhine hypodermically, and an additional one-quarter of a grain was administered five minutes before the operation was begun. During the course of the operation, which lasted one hour and forty minutes, an additional eighth of a grain was given. The patient was entirely free from pain throughout. The antidotes to morphine poisoning (atropine sulphate, gr. $\frac{1}{100}$, Strychnine suphate, gr. $\frac{1}{20}$) should be in hypodermic syringes ready for use, and strong coffee for rectal injection."—*Medical Review of Reviews.*

